# Editorial: Roles of Regulatory RNAs in Bacterial Pathogens

**DOI:** 10.3389/fmicb.2022.915005

**Published:** 2022-06-15

**Authors:** Florence Hommais, Olga Soutourina

**Affiliations:** ^1^Université de Lyon, Université Claude Bernard Lyon 1, INSA-Lyon, CNRS, UMR5240 MAP, Villeurbanne, France; ^2^Université Paris-Saclay, CEA, CNRS, Institute for Integrative Biology of the Cell (I2BC), Gif-sur-Yvette, France; ^3^Institut Universitaire de France (IUF), Paris, France

**Keywords:** metabolism, physiology and virulence control, diversity of regulatory RNAs, RNA-based mechanisms, plant, human, animal pathogens, host-pathogen interactions

Since the discovery of the first regulatory RNAs in bacteria, the amount of data on potential RNA regulators has been consistently increasing, particularly over the last couple of years. Recent technical advances have led to the identification of a large number of highly diverse regulatory RNAs, as well as their potential targets. While not all of their biological roles and molecular mechanisms of action are known, we have begun to appreciate their importance in regulatory networks governing bacterial physiology and infectious processes. In bacterial pathogens, non-coding RNAs have been recently identified as central players reprogramming gene expression in response to environmental constraints in order to adapt bacterial physiology and metabolism to host conditions. This Research Topic is focused on the role of regulatory RNAs from bacterial pathogens and is dedicated to the memory of Brice Felden and his contributions to the field of bacterial small RNAs (sRNAs). We have collected 12 excellent papers encompassing 6 original research articles and 6 reviews covering different aspects of RNA-based regulation in pathogens, which belong to both Gram-positive and Gram-negative bacteria. While many of them are focusing on human and animal pathogens including extracellular (*Helicobacter pylori, Yersinia pseudotuberculosis, Pseudomonas aeruginosa, Bacillus anthracis*), intracellular (*Listeria monocystogenesis*), and opportunistic pathogens (*Staphyloccocus aureus, Streptococci*), one study also presents results on a phytopathogenic bacterium (*Dickeya dadantii*).

A review by Felden and Augagneur provides a good overview of the diversity of pathophysiological and metabolic processes targeted by RNAs and the diversity of the RNAs themselves in terms of their genomic location, biogenesis, and mode of action. Recent studies extended the boundaries of regulatory RNA classes with novel mechanisms of action and biogenesis that are constantly discovered. They suggest a broad definition of bacterial sRNA as any RNA molecule that interacts with other actors to regulate gene expression.

Four review papers in this Research Topic are devoted to riboregulation in a particular pathogen with a specific focus on Gram-positive pathogens for three of them. First, Tejada-Arranz and De Reuse covered different aspects of RNA-based regulatory mechanisms in an important Gram-negative gastric pathogen *H. pylori*. With only a few transcriptional regulators described in this pathogen, the posttranscriptional level occupies a major but original position in the control of gene expression for efficient colonization of the hostile acidic environment inside the stomach. The absence of well-described Hfq and ProQ chaperones suggests that *H. pylori* do not follow the general rules established in model bacteria like *Escherichia coli* and *Salmonella*. The authors also highlight the role of riboregulation in persister formation via toxin-antitoxin mechanisms and in phase variation.

Second, the review by Krawczyk-Balska et al. summarizes 20 years of research on the RNA-mediated regulation of virulence and metabolism in *L. monocytogenes* starting from the identification of the thermosensor at the 5′UTR of the gene-encoding master regulator of virulence, PrfA. A number of unique regulatory mechanisms were discovered in this pathogen including rare examples among Gram-positive bacteria of RNA chaperone Hfq-associated RNAs (LhrA, LhrB, and LhrC1-5), the first evidence for riboswitch-derived sRNA SreA acting *in trans* to inhibit *prfA* mRNA translation, new dual-function long antisense RNAs named excludons acting both as *cis*-antisense RNAs and mRNAs, and finally, the secreted RNAs that modulate the innate immune response in the host through the interaction with the RNA sensor RIG-I.

In *S. aureus*, regulatory RNAs are placed together with transcriptional regulators at the center of the regulatory network ensuring successful adaptation of this opportunistic pathogen to various infection sites. Besides the well-described RNA III, the review by Barrientos et al. assembles the pieces of the puzzle from our current knowledge of riboregulation governing metabolism, stress responses, and virulence. The authors discussed the future challenges to fill the gaps in this regulatory puzzle to understand the significance of recent data on different sRNA interconnections and to identify new RNA-binding proteins important for RNA metabolism and action in *S. aureus*.

In addition to these reviews, several investigators in this Research Topic addressed the role of sRNAs in complex regulatory networks orchestrating the control of virulence factor expressions during the different stages of bacterial infection. They highlighted the interconnection of transcriptional regulation by transcriptional factors and post-transcriptional regulation by sRNAs. The role of sRNAs controlled by the two-component system CiaRH in bacterial adaptation, virulence, and resistance to antibiotics and to the host immune system is discussed in the review by Jabbour and Lartigue. These sRNAs are conserved in the *Streptococcus* genus. The authors present a comprehensive inventory for this class of sRNAs, named csRNAs, and addressed open questions on the presence and role of multiple homologous sRNAs in the CiaRH regulons. Among the original research articles, Corsi et al. characterized two regulatory sRNAs whose expression is dependent upon the master virulence regulator of *B. anthracis* AtxA, and Böhme et al. demonstrated the control of the sRNA CsrC by YmoA, a member of the Hha family in *Y. pseudotuberculosis*. The expression of virulence factors is in close association with stress adaption. In this area, Ferrara et al. characterized an sRNA named ErsA, suggesting its role in the regulation of the master regulator of anaerobiosis in *P. aeruginosa*, whereas Scheller et al. described an RNA thermometer in the 5′UTR of the *Y. pseudotuberculosis ompA* transcript involved in the host-body temperature-dependent control of this outer membrane protein known as an important virulence factor. Finally, the study by Leonard et al. on *D. dadantii* strains defective for the RNA chaperones Hfq and ProQ suggests that sRNAs would regulate targets during the different steps of the infection process. The lack of these well-characterized RNA chaperones in some bacterial groups or their questionable role in the majority of Gram-positive bacteria suggests the involvement of additional proteins in sRNA-based regulations (Tejada-Arranz and De Reuse; Barrientos et al.; Felden and Augagneur).

The RNA-mediated host-pathogen crosstalk could even be deeper. Li and Stanton discuss the underappreciated role of extracellular vesicles (EVs) for the transfer of sRNAs, especially tRNA fragments, and the role of EVs in the inter- and intra-kingdom communications without direct cell-cell contact. Based on results obtained in *P. aeruginosa* and *H. pylori*, the authors propose that tRNA fragment products delivered into the host cells by EVs would allow them to hijack the host immune response to enhance pathogen survival and that this mechanism would be widespread. Indeed, recent studies explored the secRNome of major Gram-negative and Gram-positive pathogens including secreted RNAs and vesicle-derived RNAs that could modulate the host immune response as a new mechanism for host-pathogen interactions (Lécrivain and Beckmann, 2020).

In addition to house-keeping tRNAs as a potential source of regulatory RNAs (Lalaouna et al., 2015; Li and Stanton), Stenum et al. described three sRNAs named RrA, RrB, and RrF generated from tRNA-linked repeats located downstream from the 5S gene within the ribosomal operons *rrnA, rrnB*, and *rrnD* in *E. coli*. Despite modest phenotypic effects of deletion or overexpression of these sequences in *E. coli*, their conservation in different Enterobacteria, including pathogenic species, suggests a role for these sRNAs in the fine-tuned regulation of growth phase-related processes. In eukaryotes, together with numerous tRNA-derived fragments controlling important cellular processes, several functional miRNAs have been identified as co-transcribed with rRNA primary transcripts. Further studies will define the function of these newly described bacterial sRNAs derived from ribosomal operons.

The regulatory RNA genes can be located not only in bacterial chromosomes but also within mobile genetic elements, including prophage-, pathogenicity island- and plasmid-associated RNAs. As an example, Corsi et al. characterized two sRNAs named XrrA and XrrB encoded from the virulence pXO1 plasmid of *B. anthracis*. These sRNAs are highly conserved upon closely related species carrying pXO1-like plasmids, and despite their plasmid loci, these regulatory RNAs control multiple target genes on the chromosome, placing these RNAs at the center of the crosstalk between genetic elements of *B. anthracis*. This is the second example of plasmid-transcribed regulatory sRNA controlling chromosomal-encoded targets relating to virulence suggesting such regulation could be widespread.

## Concluding Remarks and Perspectives

All original research and review articles in this Research Topic highlight the importance of RNA-based mechanisms in bacterial pathogens. Numerous new facets of these regulations shaping the adaptive and pathogenic processes both in the bacterium and in its host remain to be explored, generating several directions for future research. Among them, future studies should address questions about the roles of RNAs in the regulatory crosstalk of bacterial pathogens with their hosts and within complex microbial communities focusing on the interactions of sRNAs with both the bacterial and host RNA-binding proteins, the role of RNAs in persister formation, in phase variation, and in the interplay between mobile genetic elements, and the bacterial chromosomes that question evolutionary aspects of regulatory RNA transfer and maintenance. Furthermore, regulatory RNA integration into complex genetic circuits and impact of cellular compartmentation on sRNA regulation should be investigated. Such accumulating knowledge should provide an excellent basis for innovative therapeutic approaches targeting both protein and RNA components of regulatory networks to control pathogen development and dissemination of adaptive traits within bacterial populations.

## Author Contributions

FH and OS wrote the manuscript. Both authors contributed to the article and approved the submitted version.

## Funding

This work was supported by grants from the University of Lyon 1, the CNRS, the University Paris-Saclay, the Institute for Integrative Biology of the Cell, the DIM-1HEALTH regional Ile-de-France program (LSP Grant No. 173403), the CNRS-RFBR PRC 2019 (Grant Nos. 288426 and 19-54-15003), and the Institut Universitaire de France.

## Conflict of Interest

The authors declare that the research was conducted in the absence of any commercial or financial relationships that could be construed as a potential conflict of interest.

## Publisher's Note

All claims expressed in this article are solely those of the authors and do not necessarily represent those of their affiliated organizations, or those of the publisher, the editors and the reviewers. Any product that may be evaluated in this article, or claim that may be made by its manufacturer, is not guaranteed or endorsed by the publisher.
